# Risk Factors for Buruli Ulcer: A Case Control Study in Cameroon

**DOI:** 10.1371/journal.pntd.0000101

**Published:** 2007-12-19

**Authors:** Régis Pouillot, Gonçalo Matias, Christelle Mbondji Wondje, Françoise Portaels, Nadia Valin, François Ngos, Adelaïde Njikap, Laurent Marsollier, Arnaud Fontanet, Sara Eyangoh

**Affiliations:** 1 Laboratoire d'Epidémiologie et de Santé Publique, Centre Pasteur du Cameroun, Yaoundé, Cameroon; 2 Unité d'Epidémiologie des Maladies Emergentes, Institut Pasteur, Paris, France; 3 Laboratoire des Mycobactéries, Centre Pasteur du Cameroun, Yaoundé, Cameroon; 4 Mycobacterium Unit, Institute of Tropical Medecine, Antwerp, Belgium; 5 Hôpital de District d'Akonolinga, Ministère de la Santé Publique, Yaoundé, Cameroon; 6 Médecins Sans Frontières-Suisse, Yaoundé, Cameroon; 7 Groupe d'Etude des Interactions Hôtes Parasites, Université d Angers, Angers, France; 8 Equipe Avenir Institut National de la Santé et de la Recherche Médicale, Institut Pasteur Korea, Seongbuk-gu, Seoul, Korea; University of Tennessee, United States of America

## Abstract

**Background:**

Buruli ulcer is an infectious disease involving the skin, caused by *Mycobacterium ulcerans*. This disease is associated with areas where the water is slow-flowing or stagnant. However, the exact mechanism of transmission of the bacillus and the development of the disease through human activities is unknown.

**Methodology/Principal Findings:**

A case-control study to identify Buruli ulcer risk factors in Cameroon compared case-patients with community-matched controls on one hand and family-matched controls on the other hand. Risk factors identified by the community-matched study (including 163 pairs) were: having a low level of education, swamp wading, wearing short, lower-body clothing while farming, living near a cocoa plantation or woods, using adhesive bandages when hurt, and using mosquito coils. Protective factors were: using bed nets, washing clothes, and using leaves as traditional treatment or rubbing alcohol when hurt. The family-matched study (including 118 pairs) corroborated the significance of education level, use of bed nets, and treatment with leaves.

**Conclusions/Significance:**

Covering limbs during farming activities is confirmed as a protective factor guarding against Buruli ulcer disease, but newly identified factors including wound treatment and use of bed nets may provide new insight into the unknown mode of transmission of *M. ulcerans* or the development of the disease.

## Author Summary

Buruli ulcer (BU) is a neglected tropical infectious disease caused by *Mycobacterium ulcerans*. While BU is associated with areas where the water is slow-flowing or stagnant, the exact mechanism of transmission of the bacillus is unknown, impairing efficient control programs. Two hypotheses are proposed in the literature: previous trauma at the lesion site, and transmission through aquatic insect bites. Using results from a face-to-face questionnaire, our study compared characteristics from Cameroonian patients with Buruli ulcer to people without Buruli ulcer. This latter group of people was chosen within the community or within the family of case patients. The statistical analysis confirmed some well-known factors associated with the presence of BU, such as wearing short lower-body clothing while farming, but it showed that the use of bed nets and the treatment of wounds with leaves is less frequent in case patients. These newly identified factors may provide new insight into the mode of transmission of *M. ulcerans*. The implication of domestic or peridomestic insects, suggested by the influence of the use of bed nets, should be confirmed in specific studies.

## Introduction

Buruli ulcer (BU) is an infectious disease involving the skin, caused by *Mycobacterium ulcerans*, characterized by a painless nodule, papule, plaque or edema, evolving into a painless ulcer with undermined edges, often leading to disabling sequelae [1]. BU has been reported from 30 countries in Africa, the Americas, Asia and the Western Pacific, mainly in tropical and subtropical regions [2],[3]. The epidemiologic pattern is defined by the presence of confined foci where BU is endemic [1],[3], with prevalence ranging from a few cases to up to 22% in given communities [4]. The preventive and therapeutic tools for reducing the impact of this disease are still very limited [5],[6].

**Figure 1:**
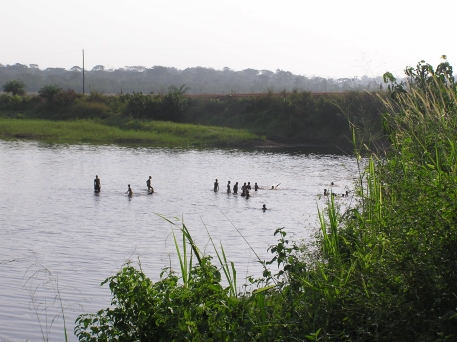
People having a bath for leisure in the Nyong River near Akonolinga (Cameroon).

In Cameroon, BU was first described in 1969 in 47 patients in a well confined area located in the neighborhood of the villages of Ayos and Akonolinga (“Province du Centre”), in the valley of the Nyong river [7]. The Nyong river basin in this area is characteristically known for its swampy banks (Figure 1). Cocoa and coffee farming was the main resource activity until the international pricing crisis of the 1990s. Known in this area as “Atom”, this disease did not arouse particular interest among public health professionals until the beginning of this century, when BU was “rediscovered” [8]. A cross-sectional study in the Nyong river basin in 2001 identified 436 patients with active or inactive BU, giving an estimated prevalence of 0.44% [8]. It is unclear whether BU has re-emerged or if cases had been undiagnosed due to the fear of stigmatization [9]–[11].

Buruli ulcers are associated with areas where the water is slow flowing or stagnant [1], [12]–[15]. Ecologic transformations have been frequently associated with occurrence or increase in BU incidence [16]. Nevertheless, the exact mechanism of transmission of the mycobacterium and the development of the disease through water-related human activities is unknown. Previous trauma at the lesion site has been recognized as a route of infection [17]. More recently, insects have been suggested to be involved in transmission of *M. ulcerans*, either through bites or by contamination of a previous trauma site [18]. This hypothesis is supported by experimental evidence showing that *M. ulcerans* can be transmitted to laboratory mice by the bite of aquatic bugs (*Naucoridae*) infected with this organism [19].

Few case-control studies have been published [4], [12], [20]–[24] and none of these concern Cameroon. We conducted a case-control investigation in Cameroon seeking for environmental and behavioral risk factors for BU, with two categories of controls: *^i^*) an age- and community-matched control and *ii*) a family-matched control.

## Methods

### Study design and case definitions

A double-matched case-control study was designed in the two health districts of Cameroon where BU is endemic, i.e. Akonolinga and Ayos.

A probable case of BU was defined as a patient presenting with active or inactive BU [1] in one of the two BU treatment centers in the area. The clinical diagnosis of BU was made by trained and specialized health practitioners in charge of the BU treatment centers. A confirmed case was defined as a probable case with evidence of *M. ulcerans* infection, indicated by a Ziehl-Neelsen test for acid-fast bacilli in smears of lesion exudates [25], a positive polymerase chain reaction (PCR) [26] or both. Laboratory analyses were done at the mycobacteria reference laboratory in Centre Pasteur du Cameroun, Yaoundé, Cameroon.

An eligible control was defined as a person who had no signs or symptoms of active or inactive BU. One age- and village-matched control was selected. A control child for child case-patients attending primary school was randomly sampled within the same classroom. Controls for other children and adult case-patients were randomly sampled within the village. An unaffected member of the family of each patient was enrolled as a family-matched control (formally: the nearest brother/sister in age). No family-matched control was enrolled when the patient was a single child or when his/her siblings lived out of the study area.

Study enrollment was voluntary. Written informed consent was obtained from case-patients and control subjects or from their parents or guardians. All BU case-patients had received or were currently receiving free treatment for BU in one of the two BU treatment centers. The study protocol was approved by the National Ethics Committee and the Cameroon Ministry of Public Health.

### Sample size

The sample size for matched case-control studies [27] was evaluated 168 pairs of one case patient/one control (control case ratio 1, odds ratio ≥2, power (1-β) 0.8, significance level (α) 0.05, correlation of exposure between pairs in the case-control set (ϕ) 0.2, calculated using the SAMPSI_MCC Stata (Stata Corporation) module [28])

### Data Collection

In February and March 2006, study personnel administered two standard questionnaires to participants concerning demographic, environmental and behavioral risk factors (see supporting information file). The first questionnaire concerned familial items (e.g. house characteristics and environment) and was given to each case-patient and his/her matched community control. The second questionnaire concerned individual items (e.g. activity and personal exposure to water) and were responded to by all case-patients and controls. All questions were close-ended. Questionnaires were verbally administered in French and/or in Ewondo (the local language). Both languages are regularly spoken irrespective to educational level of inhabitants. Case-patients were interviewed about their habits the year before onset of symptoms; controls were interviewed about their habits the year before the interview.

### Statistical methods

Community-matched and family-matched case-control studies were analyzed independently. Univariate and multivariate conditional logistic regressions were used to assess the link between variables and BU within the matched group of one case-patient/one control, using the R software (The R Core Team [29], “clogit” function, “survival” library).

Following the univariate analysis, variables that attained a p-value<0.10 were retained for multivariable analysis. A procedure using backward and forward selection based on the Likelihood Ratio Test (LRT) was used to obtain the final model.

The same initial multivariate model, excluding familial items, was used for the intra-familial case-control study, followed by the same algorithm, based on the LRT, for selection of variables.

## Results

We enrolled 163 probable cases, 163 community-matched controls and 118 familial controls.

### Characteristics of Case patients

Among the 163 probable cases, 111 (68%) were confirmed by a positive PCR. Six additional probable cases (4%) were confirmed by a single positive Ziehl-Neelsen test. The remaining probable case-patients had not been sampled for BU confirmation when symptoms were present and no sampling could be done at the time of the study as the lesions had healed. No significant difference was observed between probable cases and confirmed cases in terms of demographic data, type of first lesion, and localization of lesion (Table 1). Probable cases and confirmed cases were combined for the main analyses of the study and supplementary analyses using confirmed case-control pairs was done to corroborate results obtained from the whole dataset.

**Table 1:** Characteristics of total cases, probable cases and confirmed cases.

Characteristics	Total cases (n, %)	Probable cases (n, %)	Confirmed cases (n, %)	p*
n	163 (100)	46 (28)	117 (72)	
Sex				
Female (n, %)	79 (48)	18 (39)	61 (52)	0.16†
Male (n, %)	84 (52)	28 (61)	56 (48)	
Age (median, range)	14 (1–78)	15.5 (1–74)	13 (1–78)	0.30† ‡
<10	45 (28)	14 (3)	31 (27)	0.27†
[10]–[15]	41 (25)	7 (15)	34 (29)	
[15]–[24]	38 (23)	11 (24)	27 (23)	
≥24	39 (24)	14 (30)	25 (21)	
First lesion§				0.10†
papule	31 (19)	10 (22)	21 (18)	
nodule	60 (37)	11 (24)	49 (42)	
plaque	20 (12)	5 (11)	15 (13)	
edema	46 (28)	17 (37)	29 (25)	
active ulcers	5 (3)	3 (7)	2 (2)	
Localization¶				
Leg	92 (58)	28 (62)	64 (56)	0.85†
Arm	57 (36)	14 (31)	43 (38)	
Trunk	7 (4)	2 (4)	5 (4)	
Head	3 (2)	1 (2)	2 (2)	
Distal	103 (65)	31 (69)	72 (63)	0.58†
Proximal/Trunk/Head	56 (35)	14 (31)	42 (37)	
Right side	57 (38)	14 (31)	43 (38)	0.46†
Left side	92 (61)	28(62)	64 (56)	

*: Probable *vs*. confirmed cases, Fisher's exact test;

**†:** Nonsignificant;

**‡:** two sample t-test;

**§:** one missing piece of data;

**¶:** four missing pieces of data

The median age of all recruited patients was 14 years (range: 1 to 78 years). However, male case-patients were generally younger than female case-patients (median age: 12 and 19, respectively; p<0.01, nonparametric K-sample test for equality of medians).

When interviewed, 25 patients had contracture deformities or scars and three had had an amputation. The first BU lesions in most cases occurred on the leg (92/159, 58%, data missing for 4 cases) or arm (57/159, 36%). Initial lesions occurred less frequently on the trunk (7/159, 4%) and head (3/159, 2%). More patients had lesions on a distal extremity (from the elbow to the hand and from the knee to the foot, 103/159, 65%) than on a proximal extremity, trunk or head (56/159, 35%, Fisher exact test: p<0.01). When first BU lesions appeared on a limb, it was more frequently on the lower limbs than upper limbs (92 and 57, respectively; Fisher exact test: p = 0.01) and more frequently on the left side than on the right side (92 and 57, respectively; Fisher exact test: p = 0.01). There was no significant difference in this distribution of lesions associated with the sex or the age (<10 compared to >10 years) of case-patients.

Most case-patients (127/163, 76%) did not declare an association between the occurrence of their first lesion and a particular event. Nevertheless, 16/163 (10%) associated it with an injury, 15/163 (9%) with an insect bite and 8/163 (5%) with another event. While all case-patients had been treated in a hospital, as recruitment was hospital-based, 80/162 had been treated in parallel or consecutively by a traditional practitioner.

### Case-/Community-matched control

#### Univariate Analysis (Table 2)

**Table 2:** Univariate analysis of selected variables for Buruli ulcer disease in Cameroon, Community-matched case-control study.

Characteristic	No. (%) of control subjects (n = 163)	No. (%) of case-subjects (n = 163)	Univariate OR (95% CI) *	p †
*Demographic*				
Ethnic group of the father: Maka/others	13 (8)	22 (14)	2.0 (0.90–4.5)	0.08
Ethnic group of the mother: Maka/others	14 (9)	16 (10)	1.2 (0.53–2.6)	0.68
Education level: Primary or any/Secondary or more	94 (58)	121 (74)	4.0 (1.9–8.3)	<0.01
*Economic level*				
* *Household spends<than 1 €/day/≥1 €/day‡	52 (32)	62 (38)	1.6 (0.86–2.8)	0.14
*Health*				
BCG scar: Yes/No	114 (70)	96 (59)	0.61 (0.38–0.97)	0.04
History of Tuberculosis: Yes/No	6 (4)	11 (7)	1.8 (0.67–5.0)	0.22
Family history of tuberculosis: Yes/No	17 (10)	14 (9)	1.2 (0.59–2.6)	0.57
Ever had blood in urine: Yes/No	5 (3)	5 (3)	1.0 (0.25–4.0)	1.0
*Household Environment*				
Mud wall: Yes/No	59 (36)	65 (40)	1.2 (0.74–1.8)	0.49
Mud floor: Yes/No	74 (45)	82 (50)	1.3 (0.78–2.0)	0.33
Within 15 min vs>15 min of the Nyong river	13 (8)	24 (15)	2.2 (1.0–4.9)	0.04
No. of people in household: >8 vs ≤ 8	71 (44)	78 (48)	1.2 (0.8–1.9)	0.42
Cocoa plantation in the immediate neighborhood: Yes/No	50 (31)	72 (44)	1.8 (1.1–2.8)	0.01
Coffee plantation in the immediate neighborhood: Yes/No	66 (40)	83 (51)	1.6 (1.0–2.5)	0.05
Bush in the immediate neighborhood: Yes/No	112 (69)	119 (73)	1.2 (0.76–2.0)	0.38
Woods in the immediate neighborhood: Yes/No	7 (4)	18 (11)	3.2 (1.2–8.7)	0.01
Swamp in the immediate neighborhood: Yes/No	21 (13)	45 (28)	2.7 (1.5–5.0)	<0.01
River in the immediate neighborhood: Yes/No	20 (12)	30 (18)	1.6 (0.87–3.0)	0.12
Share living space with goats: Yes/No	49 (30)	69 (42)	1.7 (1.1–2.8)	0.02
Share living space with poultry: Yes/No	116 (71)	127 (78)	1.6 (0.89–2.8)	0.11
Share living space with pigs: Yes/No	50 (31)	70 (43)	1.8 (1.1–3.0)	0.02
Share living space with cats: Yes/No	77 (47)	94 (58)	1.6 (1.0–2.5)	0.05
Share living space with dogs: Yes/No	62 (38)	77 (47)	1.4 (0.93–2.2)	0.10
*Primary source of drinking water*				
Network	39 (24)	37 (23)	1 (reference)	
River or stream	81 (50)	69 (42)	0.88 (0.46–1.7)	0.70
Borehole	43 (26)	57 (35)	1.4 (0.72–2.7)	0.32
*Insect bites/behavior*				
Received insect bite in water/mud: Yes/No	48 (29)	70 (43)	1.8 (1.1–2.8)	0.01
Use bed nets: No/Yes	51 (31)	74 (45)	2.0 (1.2–3.4)	<0.01
Use mosquito coils: Yes/No	84 (51)	110 (67)	2.1 (1.3–3.4)	<0.01
*Treatment when hurt*				
Soap and water: Yes/No	36 (22)	57 (35)	1.9 (1.1–3.1)	0.01
Rubbing alcohol: No/Yes	85 (52)	120 (74)	2.8 (1.6–4.6)	<0.01
Leaves: No/Yes	138 (84)	153 (94)	2.7 (1.2–5.7)	0.01
Adhesive bandage: Yes/No	21 (13)	50 (31)	3.4 (1.8–6.5)	<0.01
*Activities*				
Waded in the Nyong swamp: Yes/No	10 (6)	35 (21)	5.1 (2.2–12)	<0.01
Waded in a river or stream: Yes/No	5 (3)	21 (13)	5.0 (1.7–15)	<0.01
*Activities*				
* *Wash clothes: No/Yes	16 (10)	34 (21)	2.6 (1.3–5.3)	0.01
Fetch water: Yes/No	136 (83)	132 (81)	0.82 (0.45–1.5)	0.53
Farm: Yes	132 (81)	136 (83)	1.2 (0.66–2.3)	0.40
Do not farm	31 (19)	27 (17)	1 (reference)	
Farm and wear long upper body clothing/shirt	89 (55)	85 (52)	1.1 (0.59–2.2)	0.38
Farm and wear short upper body clothing/shirt	43 (26)	51 (31)	1.4 (0.70–2.9)	0.98
Do not farm	31 (19)	27 (17)	1 (reference)	
Farm and wear long pants/dress	117 (72)	104 (64)	1.0 (0.50–2.0)	0.91
Farm and wear short pants/dress	15 (9)	32 (20)	2.5 (1.1–5.8)	0.03
Fish: Yes/No	54 (33)	76 (47)	2.0 (1.2–3.3)	0.01
Do not fish	109 (67)	87 (53)	1 (reference)	
Fish, but not in the Nyong river	40 (25)	40 (25)	1.3 (0.73–2.4)	0.36
Fish in the Nyong river	14 (9)	36 (22)	4.5 (1.9–10)	<0.01
Do not fish	109 (67)	87 (53)	1 (reference)	
Fish with long upper body clothing	38 (23)	44 (27)	1.7 (0.92–2.9)	0.09
Fish with short/no upper body clothing	16 (10)	32 (20)	2.8 (1.4–5.9)	0.01
Do not fish	109 (67)	87 (53)	1 (reference)	
Fish with long lower body clothing	42 (26)	59 (36)	2.1 (1.2–3.6)	0.01
Fish with short/no lower body clothing	12 (7)	17 (10)	1.9 (0.83–4.2)	0.12
*Bath (hygiene)*				
Have bath for hygiene: Yes/No	57 (53)	116 (71)	2.3 (1.4–3.8)	<0.01
Do not have bath for hygiene	76 (47)	47 (29)	1 (reference)	
Have bath for hygiene, but not in the Nyong river	67 (41)	76 (47)	1.8 (1.0–3.1)	0.04
Have bath for hygiene in the Nyong river: Yes	20 (12)	40 (25)	4.7 (2.0–11)	<0.01
Do not have bath for hygiene	76 (47)	47 (29)	1 (reference)	
Have bath for hygiene, but not in open borehole	71 (44)	80 (49)	2.0 (1.2–3.4)	0.01
Have bath for hygiene in open borehole	16 (10)	36 (22)	3.7 (1.8–7.6)	<0.01
*Swim/dive/play in water*				
Swim: Yes/No	80 (49)	98 (60)	1.75 (1.1–2.9)	0.02
Do not swim	83 (51)	65 (40)	1 (reference)	
Swim, but not in the Nyong river	57 (35)	51 (31)	1.2 (0.69–2.1)	0.50
Swim in the Nyong river	23 (14)	47 (29)	4.8 (2.0–11)	<0.01
*Beliefs on the origin of BU*				
wound: Yes/No	2 (1)	6 (4)	3.0 (0.61–15)	0.15
insect bites: Yes/No	15 (9)	6 (4)	0.4 (0.16–1.0)	0.05
witchcraft: Yes/No	45 (28)	55 (34)	1.3 (0.83–2.1)	0.23
do not know: Yes/No	69 (42)	81 (50)	1.5 (0.89–2.4)	0.13

*: conditional logistic regression;

**†:** Wald test, conditional logistic regression;

**‡:** data missing for 84 subjects

We could only assess bacillus Calmette Guérin (BCG) vaccination by the presence of a scar, as vaccination records were generally missing. A scar was more frequently observed in the control population (Conditional logistic regression as in all the following text: p = 0.04). Few subjects reported a personal history or family history of tuberculosis. This was not significantly associated with BU. We assessed history of schistosomiasis based on the self-declaration of history of blood in the urine. Few subjects reported having a history of blood in urine, and this was not significantly associated with BU.

Based on the questionnaire about household environment, case-patients more frequently lived closer to the Nyong river than the median distance calculated for the studied population (p = 0.04). They also lived in the immediate neighborhood of a cocoa plantation (p = 0.01), a swamp (p<0.01) and/or woods (p = 0.01). Whereas significantly more case-patients than controls reported sharing living space with goats (p = 0.02) and/or pigs (p = 0.02), there was no difference between these subjects regarding reports of sharing living space with domestic carnivores or poultry.

There was no significant difference between case-patients and controls with respect to the source of drinking water, i.e. water network, river or stream or borehole water. Cases declared that they washed clothes less frequently than controls (p = 0.01), and fetching water was not significantly associated with BU.

Case-patients reported that they had been bitten by insects more frequently than controls while they were in water or wading in mud (p = 0.01). They reported use of bed nets less frequently than controls (p<0.01) and that they used mosquito coils instead (p<0.01). Note that these two variables were negatively associated (p<0.01), indicating that people use preferably one of these two method to prevent insect bites.

Whereas case-patients more frequently used soap, water (p = 0.01) and adhesive bandages when hurt (p<0.01), controls more frequently used rubbing alcohol (p<0.01) and leaves (p = 0.01). The use of adhesive bandages was negatively associated with the use of leaves (p = 0.01) and the use of soap and water was negatively associated with the use of rubbing alcohol (p = 0.03).

Wading in the Nyong river swamp and wading in a river or a stream, though less frequent, was significantly associated with case-patients (p<0.01 in both situations).

Almost all subjects reported farming activity (this variable being understood as: “follow his parents during farming activities” for children). This activity was not significantly associated with BU (p = 0.40). Nevertheless, wearing short pants or a short dress when farming was significantly associated with BU (univariate odds ratio (OR): 2.5; p = 0.03 compared to the reference group “do not farm”). There was no such difference associated with a particular type of upper body clothing.

Univariate analysis based on the responses to the questionnaire about water-associated activities indicated that fishing was a risk factor for BU (p = 0.01). More precisely, fishing in the Nyong river with short upper body clothing and/or with long lower body clothing (p = 0.01 compared to nonfishers) was significantly associated with BU. Case-patients were significantly more likely than controls to have a bath for hygiene purposes or for leisure (swimming/diving/playing) in the Nyong river and to have a bath for hygiene purposes in open boreholes (p<0.01).

At the end of the questionnaire, it was asked to participants their beliefs about the origin of BU. Most of them were ignorant of the origin of this disease (69/163, 43% for controls and 81/163, 50% for case patients) while a large part of the others think it could be due to witchcraft (45/163, 28% for controls and 55/163, 34% for case patients). No significant difference was observed in answers regarding this question between case and controls.

#### Multivariate analysis

Washing clothes, using bed nets, and treating wounds with rubbing alcohol or leaves were behavioral protective factors for BU in the final multivariate model (Table 3), whereas wading in the Nyong swamp, farming with short lower body clothing, using mosquito coils to prevent insect bites and using adhesive bandages to treat wounds were behavioral risk factors. The Odds Ratio (OR) associated with the factor “Farm with short pants/dress” was very high (15; 95% CI 4.2-58) with reference to the item “Do not farm or farm with long pants/dress”; it was estimated 25 (95% CI 4.5-140; p<0.01) with reference to “Do not farm”, using the same other variables. Living in the immediate neighborhood of cocoa plantations and/or woods were indicated to be risk factors. Lastly, less than a secondary school level of education was more frequently associated with case-patients.

**Table 3:** Multivariable model for risk factors for Buruli ulcer disease in Cameroon, Community-matched case-control study

Risk factor	Multivariate OR *(95% CI)	p †
*Demographic and activities*		
Education level: Primary or any/Secondary or more	3.6 (1.3–9.8)	0.014
Wash clothes: No/Yes	5.1 (1.5–17)	0.008
Wade in the Nyong swamp: Yes/No	5.7 (1.6–20)	0.007
*Farming activities and clothing*		
Do not farm or farm with long pants/dress	1 (reference)	
Farm with short pants/dress	15 (4.2–58)	<0.001
*Insect bites/behavior*		
Use bednets: No/Yes	2.6 (1.2–6.0)	0.022
Use mosquito coils: Yes/No	4.5 (1.8–11)	0.001
*Household environment*		
Cocoa plantation in the immediate neighborhood: Yes/No	3.2 (1.5–7.0)	0.004
Woods in the immediate neighborhood: Yes/No	6.3 (1.1–36)	0.039
*Treatment when hurt*		
Use adhesive bandage: Yes/No	6.4 (2.2–19)	0.001
Use rubbing alcohol: No/Yes	2.2 (1.0–4.6)	0.040
Use leaves: No/Yes	4.4 (1.4–13)	0.009

*: multivariate conditional logistic regression;

**†:** Wald test, multivariate conditional logistic regression

The analysis on confirmed case-control pairs only (117 pairs) corroborates that washing clothes, using bed nets, treating wounds with rubbing alcohol were protective factors for BU whereas wading in the Nyong swamp, farming with short lower body clothing and using adhesive bandages to treat wounds were confirmed as risk factors. Swimming in the Nyong river is a significant risk factor for BU in this analysis.

### Case-/Familial-matched control

#### Univariate Analysis

Median age and sex were not different between case-patients and family-matched controls. Substantially fewer factors were significant in the univariate analysis of this family-matched case-control study than in the community-matched case-control study (Table 4). A low education level was significantly associated with BU cases (p = 0.04). Those who never used bed nets were significantly more affected than those who sometimes or frequently used bed nets (p<0.01).

**Table 4:** Univariate analysis of selected variables for Buruli ulcer disease in Cameroon, Familial-matched case-control study.

Characteristic	No. (%) of control subjects (*n* = 118)	No. (%) of case subjects (*n* = 118)	Univariate OR (95% CI)*	p †
*Economic level*				
Education level: Primary or any/Secondary or more	80 (68)	91 (77)	2.4 (1.0–5.4)	0.04
*Health*				
BCG scar: Yes/No	84 (71)	71 (60)	0.62 (0.36–1.1)	0.08
History of Tuberculosis: Yes/No	6 (5)	8 (7)	1.4 (0.44–4.4)	0.56
Ever had blood in urine: Yes/No	6 (5)	3 (3)	0.40 (0.078–2.1)	0.27
*Insect bites/behavior*				
Received insect bite in water/mud: Yes/No	42 (36)	45 (38)	1.2 (0.60–2.4)	0.60
Use bed nets: No/Yes	36 (34)	56 (47)	4.3 (1.8–11)	<0.01
Use mosquito coils: Yes/No	77 (65)	81 (68)	1.2 (0.67–2.0)	0.57
*Treatment when hurt*				
Soap and water: Yes/No	37 (31)	42 (36)	1.2 (0.70–2.2)	0.47
Rubbing alcohol: No/Yes	81 (69)	82 (69)	1.0 (0.58–1.9)	0.87
Leaves: No/Yes	103 (87)	108 (92)	2.7 (0.71–10)	0.15
Adhesive bandage: Yes/No	29 (25)	37 (32)	1.5 (0.83–2.7)	0.18
*Activities*				
Waded in the Nyong swamp: Yes/No	20 (17)	21 (18)	1.1 (0.45–2.7)	0.81
Waded in a river or stream: Yes/No	52 (44)	53 (44)	1.0 (0.60–1.8)	0.89
* *Wash clothes: No/Yes	24 (20)	23 (19)	0.92 (0.40–2.1)	0.83
Fetch water: Yes/No	98 (83)	100 (84)	1.18 (0.52–2.6)	0.68
Farm: Yes/No	103 (88)	99 (84)	0.60 (0.22–1.7)	0.32
Do not farm	15 (13)	19 (16)	1 (reference)	
Farm and wear long upper body clothes/shirt	62 (53)	57 (48)	0.56 (0.19–1.6)	0.28
Farm and wear short upper body clothes/shirt	41 (35)	42 (36)	0.64 (0.22–1.8)	0.40
Do not farm	15 (13)	19 (16)	1 (reference)	
Farm and wear long pants/dress	82 (69)	74 (63)	0.55 (0.20–1.6)	0.26
Farm and wear short pants/dress	21 (18)	25 (21)	0.75 (0.24–2.3)	0.63
Fish: Yes/No	56 (47)	48 (41)	0.60 (0.29–1.2)	0.16
Do not fish	62 (53)	70 (59)	1 (reference)	
Fish, but not in the Nyong river	28 (24)	28 (24)	0.75 (0.33–1.7)	0.49
Fish in the Nyong river	28 (24)	20 (17)	0.44 (0.18–1.1)	0.08
Do not fish	62 (53)	70 (59)	1 (reference)	
Fish with long upper body clothing	47 (40)	35 (30)	0.48 (0.22–1.1)	0.07
Fish with short/no upper body clothing	9 (8)	13 (11)	1.1 (.38–3.0)	0.90
Do not fish	62 (53)	70 (59)	1 (reference)	
Fish with long lower body clothing	35 (30)	28 (24)	0.54 (0.24–1.2)	0.14
Fish with short/no lower body clothing	21 (18)	20 (17)	0.67 (0.29–1.5)	0.34
*Bath (hygiene)*				
Have bath for hygiene: Yes/No	81 (69)	79 (67)	0.88 (0.44–1.8)	0.72
Do not have bath for hygiene	37 (31)	39 (33)	1 (reference)	
Have bath for hygiene, but not in the Nyong river	57 (48)	52 (44)	0.79 (0.37–1.7)	0.53
Have bath for hygiene in the Nyong river	24 (20)	27 (23)	1.2 (0.45–3.1)	0.75
Do not have bath for hygiene	37 (31)	39 (33)	1 (reference)	
Have bath for hygiene, but not in open borehole	60 (51)	60 (51)	0.90 (0.44–1.8)	0.77
Have bath for hygiene in open borehole	21 (18)	19 (16)	0.80 (0.31–2.0)	0.64
*Swim/Dive/Play in water*				
Swim: Yes/No	75 (64)	66 (56)	0.69 (0.39–1.2)	0.20
Do not swim	43 (36)	52 (44)	1 (reference)	
Swim, but not in the Nyong river	48 (41)	37 (31)	0.57 (0.30–1.1)	0.10
Swim in the Nyong river	27 (23)	29 (25)	0.97 (0.43–2.2)	0.93

*: conditional logistic regression;

**†:** Wald test, conditional logistic regression.

#### Multivariate analysis

The final multivariate model (Table 5) indicates that using bed nets and using leaves to treat wounds were strong protective factors. Case-patients were more frequently associated with lower than a secondary-school level of education. Fishing in the Nyong river was determined to be a protective factor (0.28; 95% CI 0.094-0.84) using “Do not fish or fish, but not in the Nyong river” as the reference; the OR was estimated 0.30 (95% CI 0.10-.92; p = 0.04) with reference to “Do not fish” using the same other variables. Swimming, but not in the Nyong river appeared to be protective.

**Table 5:** Multivariable model for risk factors for Buruli ulcer disease in Cameroon, Familial-matched case-control study.

Risk factor	Multivariate OR *(95% CI)	p †
*Demographic*		
Education level: Primary or any/Secondary or more	4.9 (1.8–13)	0.002
*Insect bites/behavior*		
Use bed nets: No/Yes	10 (3.1–33)	<0.001
*Treatment when hurt*		
Use leaves: No/Yes	7.8 (1.6–37)	0.011
*Swim/Dive/Play in water*		
Do not swim	1 (reference)	
Swim, but not in the Nyong river	0.33 (0.15–0.72)	0.005
Swim in the Nyong river	1.0 (1.0–9.8)	0.950
*Fishing activities*		
Do not fish or fish but not in the Nyong river	1 (reference)	
Fish in the Nyong river	0.28 (0.094–0.84)	0.024

*: conditional logistic regression;

**†:** Wald test, conditional logistic regression.

The analysis on confirmed case-control pairs only (89 pairs) corroborates that a low education level was a risk factor and that using bed nets, using leaves to treat wounds and swimming were protective factors. Wearing long pants while fishing was a significant protective factor and using soap to treat wounds was a significant risk factor in this latter analysis.

## Discussion

This case-control study identifying BU risk factors in Cameroon is the first published.

### Limitations of the study

Case-control study limitations are undoubtedly applicable to this study. The study could not be done on incident cases due to the low incidence of BU in the area. Cases that were at early stages of infection could not be included which could possibly induce a bias. Memory bias may have occurred as onset of BU symptoms could have happened a long time before the study. Beliefs about BU may have modified participants' responses; nevertheless, this study confirms that most people do not have any knowledge of the origin of the disease or think it is due to witchcraft, like in other BU endemic countries [9],[10]. Also, interviewers were not blinded to the disease status of participants.

The proportion of confirmed BU cases in Cameroon is increasing, especially since the PCR technique was implemented in the Centre Pasteur du Cameroun, Yaoundé. Nevertheless, the statistical power of the analysis is lower if we restrict the study to confirmed cases. The similar characteristics of confirmed and unconfirmed cases give us confidence to combine these two subpopulations, as well as the high sensitivity of the clinical diagnostic found elsewhere [21]. The presence of misclassification (false positive cases) is not excluded. Possible misclassification of BU-free patients as case-patients reduces the statistical power of the study and may introduce bias [30]. Nevertheless, we confirmed the major factors identified on the whole dataset when analyses were done on the sub-sample of confirmed case-control pairs.

We matched case-patients with controls from their villages of residence, but did not use the nearest-neighbor method in order to avoid overmatching. Overmatching is evident in the family case-control study. Results from both analyses are not independent as the case-samples were the same. Nevertheless, the identification of a risk factor in both family and community case-control studies provides additional support for that risk factor.

### Cases

Our study design could not investigate the influence of age on BU. The majority of case-patients are children under 15 years of age, as described in other publications [1]. We confirm the prevalence of BU lesions on the extremities, especially on the legs [8],[12],[15],[31]. A study carried out in Ghana in 1989 reported that the left leg was more frequently affected than the right leg in adults [31], but this asymmetrical distribution was not confirmed in a more recent study [32] or in Cameroon [8]. We found an unequal right-left distribution in favor of the left side. We found no association between this asymmetrical distribution and sex or age; thus, we cannot make assumptions about the differential behavior within these subpopulations regarding exposure to BU infection.

### Risk factors

This is the largest case-control study using face-to-face questionnaire ever published for BU. Nevertheless, one should address the possible lack of power of the analysis to identify factors weakly related to the transmission or development of this disease. Indeed, some factors not determined to be risk factors in this study deserve comment. BCG vaccination is known to be effective against leprosy [33]. Though univariate analysis in this study indicates that it is a protective factor for BU, multivariate analysis assessing confounding factors does not confirm this finding, similar to previous reports [21],[24]. A higher risk for BU in BCG-vaccinated patients ≥5 years of age was recently observed in a case-control study on 2,399 case files [23], but we did not observe this in our study. Though fetching water has been suggested to be a risk factor for BU [34], we see no evidence of this in our study. Unlike previous reports [15],[23], our findings do not suggest that use of unprotected water sources is a significant risk factor.

A low education level of the subject (<secondary) was observed as a significant risk factor for BU in both the case-/community- and case-/familial-matched control studies. All pairs of children <12 years old having a <secondary level of education, this effect is observed only from the teenagers and the adults records. This factor was not linked to e.g. farming activities, bath or swimming activities or wound treatment.

We found that living near a cocoa plantation or woods is a risk factor for BU in this area. The people of this area made their livings from cocoa plantations until the major crisis of the mid-1990s. Many study participants noted that cocoa farmers developed food crop plantations near the Nyong river following this crisis. This development was associated with profound ecologic upheaval. Further studies including geographic information systems should be conducted, but these ecologic changes might be related to the re-emergence of BU in the area.

BU endemicity in this area is associated with the presence of the Nyong swamp. It is extremely difficult to determine risky behaviors for BU infection with more precision, as being exposed to water bodies are part of the daily routine of a majority of inhabitants of this area. Many variables linked to exposure to water, and especially to the river Nyong swamp, are significantly associated with cases of BU in univariate community-matched and family-matched analyses. Nevertheless, we observed many colinearities. The final multivariate models only show that *i*) wading in the Nyong river swamp (community-matched analysis) and swimming in the Nyong river (family-matched analysis) are risk factors, and *ii*) fishing in the Nyong river (family-matched analysis) is a protective factor. Wading in a river or stream has been identified as a risk factor in Ghana [21] and indirectly in Benin as swamp water is a primary water source [23]. Swimming was found to be a significant risk factor in Ghana [20]. Community-matched analysis did not indicate that fishing in the Nyong river, implying daily exposure to the Nyong river and its swamp, is an independent risk factor. Additionally, family-matched analysis indicated that it is a protective factor. Fishing activities have never been found to be independent risk factors for BU. We hypothesize that heavily exposed populations acquire protection against *M. ulcerans*, or at least against the *M. ulcerans*-driven pathogenic processes. Marsollier *et al.* [35] showed that prior exposure to bites from *M. ulcerans*–free aquatic insect predators confers some protection against *M. ulcerans* infection. The same hypothesis is applicable to the protective effects of washing clothes, which also implies daily exposure to water. Wound-treatment practices seem to substantially influence BU in the community-matched and the family-matched case-control studies. Use of rubbing alcohol can prevent infection of the trauma site, but the protective effect of leaves compared to adhesive bandages should be confirmed. Antiseptic or astringent active principles (e.g. tannins, flavonoids) in leaves traditionally used in these areas possibly explain this protective effect. The pharmacologic properties of leaves used traditionally in Cameroon should be investigated further.

We found that wearing long clothing during farming activities is protective against BU, as in Ghana and the Ivory Coast, [12],[21]. Note nevertheless that the wide confidence intervals obtained for this variable reflects a small amount of discordant pairs. This result may not be robust. This finding suggests that long periods of skin exposure facilitate infection. It is unusual to be bare chested in Cameroon, corroborated by the smaller number of observed lesions on the trunk than that observed in other countries [32]. This finding is consistent with both prevailing hypotheses that insect vectors and penetrating injuries are potential modes of BU transmission for *M. ulcerans* [21].

The use of bed nets is a strongly associated protective factor for BU in our study, but not in Ghana [21]. In Cameroon, bed nets are principally used to prevent malaria which is endemic in the whole country. The cost of bed nets does still not afford its universal use, and families generally do not own bed nets for the whole members of the family. Infection was less frequent among those using bed nets than those not using them, even within families. This intra-familial confirmation invalidates the hypothesis of a confounding effect linked, for example, to household location, access to bed nets or socio-economic status. The choice of the people who sleep under a bed net within a family was not investigated in this study. The impact of bed nets, especially pyrethroid-impregnated bed nets, on personal protection against the malaria mosquito is uncontroversial. Unfortunately, the questionnaire did not explore whether or not these bed nets were impregnated with insecticide. This observation is consistent with the recent detection of *M. ulcerans* by PCR in a small proportion of mosquitoes trapped in a BU-endemic area in Victoria, Australia [36],[37]. Pyrethroid impregnated bed nets also protect from other insects, including day-flying insects, crawling insects, head lice, chicken ticks or bedbugs [38]–[40]. Water bugs (genera *Naucoridae* and *Dyplonichus*), which are suspected to be a possible vector of *M. ulcerans* [18],[19] are flying insects but there common ecological area is not households. The impact of bed nets on bites from these sylvatic insects is thus less evident. Our study supports the hypothesis implicating domestic or peridomestic insects (e.g. mosquitoes) in the transmission of *M. ulcerans*.

## Conclusion

Our findings are consistent with both major hypotheses of *M. ulcerans* transmission, i.e. insect bites and/or contamination following or accompanying trauma. Treatment practices following trauma were highly significant, supporting the hypothesis involving contamination of a trauma site. However, the use of bed nets, which we propose to be a protective factor, favors the hypothesis involving an insect vector. A specific study should be undertaken to confirm these risk factors for two reasons. First, it may yield information about the mode of transmission. Second, measures to control these risks should be easy to implement to protect inhabitants from BU and other diseases.

This study confirms that wading in the swamp and wearing short clothing during farming activities are risk factors for BU. Public health messages about these risk factors can now be provided to local populations. Nevertheless, providing information, ending stigmatization, focusing on early detection and prompt treatment still make up the best public health strategy to reduce BU burden until the mode of transmission of *M. ulcerans* and the following development of the disease is more clearly understood.

## Supporting Information

Alternative Language Abstract S1 Translation of the abstract into French
(0.03 MB DOC)
